# Amyotrophic Lateral Sclerosis: A Neurodegenerative Motor Neuron Disease With Ocular Involvement

**DOI:** 10.3389/fnins.2020.566858

**Published:** 2020-09-25

**Authors:** Pilar Rojas, Ana I. Ramírez, José A. Fernández-Albarral, Inés López-Cuenca, Elena Salobrar-García, Manuel Cadena, Lorena Elvira-Hurtado, Juan J. Salazar, Rosa de Hoz, José M. Ramírez

**Affiliations:** ^1^Instituto de Investigaciones Oftalmológicas Ramón Castroviejo, Universidad Complutense de Madrid, Madrid, Spain; ^2^Hospital General Universitario Gregorio Marañón, Instituto Oftálmico de Madrid, Madrid, Spain; ^3^OFTARED, ISCIII, Madrid, Spain; ^4^Departamento de Inmunología Oftalmología y ORL, Facultad de Óptica y Optometría, Universidad Complutense de Madrid, Madrid, Spain; ^5^Departamento de Inmunología Oftalmología y ORL, Facultad de Medicina, Universidad Complutense de Madrid, Madrid, Spain

**Keywords:** ALS, motor neuron, neurodegenerative diseases, retina, optic nerve, eye, neuroinflammation, biomarker

## Abstract

Amyotrophic lateral sclerosis (ALS) is a neurodegenerative disease that causes degeneration of the lower and upper motor neurons and is the most prevalent motor neuron disease. This disease is characterized by muscle weakness, stiffness, and hyperreflexia. Patients survive for a short period from the onset of the disease. Most cases are sporadic, with only 10% of the cases being genetic. Many genes are now known to be involved in familial ALS cases, including some of the sporadic cases. It has also been observed that, in addition to genetic factors, there are numerous molecular mechanisms involved in these pathologies, such as excitotoxicity, mitochondrial disorders, alterations in axonal transport, oxidative stress, accumulation of misfolded proteins, and neuroinflammation. This pathology affects the motor neurons, the spinal cord, the cerebellum, and the brain, but recently, it has been shown that it also affects the visual system. This impact occurs not only at the level of the oculomotor system but also at the retinal level, which is why the retina is being proposed as a possible biomarker of this pathology. The current review discusses the main aspects mentioned above related to ALS, such as the main genes involved, the most important molecular mechanisms that affect this pathology, its ocular involvement, and the possible usefulness of the retina as a biomarker.

## Introduction: Overview of Amyotrophic Lateral Sclerosis Disease

Motor neuron diseases (MNDs) have a high morbidity and mortality and cause a gradual deterioration of voluntary muscle function due to progressive neuronal damage (Rowland and Shneider, 2001; Turner et al., 2009; Yedavalli et al., 2018; Pinto et al., 2019). MND has a global incidence of one to three cases per 100,000, while its prevalence ranges from one to nine cases per 100,000. Within the group of these diseases, amyotrophic lateral sclerosis (ALS) is the most common, accounting for 80–90% of all MND cases (Román, 1996) and having an incidence per 100,000 people of 0.3–2.5 cases per year (Rowland and Shneider, 2001; Sathasivam, 2010; Kiernan et al., 2011; Pratt et al., 2012). Of all ALS cases, 10% are familial (FALS) (Byrne et al., 2011) ranging from 2% to 15% depending on the population (Conwit, 2006). Overall, both the incidence (Haberlandt, 1959) and the prevalence (Williams et al., 1988) of this pathology can be variable depending on the region and ethnicity.

In ALS, a combination of the symptoms of both upper motor neurons (UMNs) and lower motor neurons (LMNs) occurs (Rowland and Shneider, 2001; Kiernan et al., 2011; Yedavalli et al., 2018). This condition involves muscle weakness and stiffness, overactive reflexes, and sometimes changes in emotions (Carra et al., 2012; Zarei et al., 2015; Yedavalli et al., 2018). ALS involves the swallowing, speech, and respiratory muscles (Carra et al., 2012; Zarei et al., 2015; Yedavalli et al., 2018). The disease usually begins in the extremities (spinal onset), although 25% of patients have a bulbar onset, which has a worse prognosis (Kiernan et al., 2011). ALS is a disease that is asymmetrical with respect to the onset and spread of UMN and LMN dysfunction and constitutes a heterogeneous disease, because not all forms behave in the same way, which makes its classification complex (Zhang et al., 2014).

Although ALS has only been considered a motor disease, neuroimaging tests have recently shown the involvement of non-motor areas such as cerebral global atrophy, decrease in gray matter density, and regional white matter alterations (Ellis et al., 2001; Abrahams et al., 2005; Kassubek et al., 2005; Ringholz et al., 2005; Mezzapesa et al., 2007; Zhang et al., 2014). The alteration of these areas leads to cognitive and behavioral changes (Abrahams et al., 2005; Mezzapesa et al., 2007). Cognitive impairment, mainly featuring executive dysfunction and mild memory loss during the course of the disease, has been found in 50% of ALS patients (Ringholz et al., 2005; Meier et al., 2010).

### Mechanisms and Risk Factors Involved in the Pathogenesis of Motor Neuron Diseases

There are different risk factors involved in ALS (Yu et al., 2014; Zarei et al., 2015), which include smoking (Weisskopf et al., 2009; Wang et al., 2014), agricultural chemicals (Ward et al., 2014; Seals et al., 2017), heavy metals (Simons, 1986; Mcguire et al., 1997; Savolainen et al., 1998; Kamel et al., 2002), and low-frequency electromagnetic waves (Zhou et al., 2012). In addition, athletes (Chiò et al., 2004; Turner et al., 2012; Zarei et al., 2015) and hypermetabolic phenotype (Desport et al., 2001; Bouteloup et al., 2009; Vaisman et al., 2009; Dupuis et al., 2011; Ferri and Coccurello, 2017; Steyn et al., 2018; Pape and Grose, 2020) have a higher risk for ALS. A low body mass index can induce disease progression and reduced survival time (Jawaid et al., 2010; Park et al., 2015). Other factors that may affect ALS patients are hyperlipidemia (Palamiuc et al., 2015) and glutamate-rich and fat-rich diets (Iwasaki et al., 2005; Veldink et al., 2007; Morozova et al., 2008; Fitzgerald et al., 2014). However, progesterone and estrogen provide protection against ALS (Gargiulo Monachelli et al., 2011; De Jong et al., 2013; Pape and Grose, 2020).

Several molecular mechanisms can cause neurodegeneration in ALS (De Vos et al., 2000; Magrané and Manfredi, 2009; Shi et al., 2010; Forsberg et al., 2011; Zhu and Sheng, 2011; Donnelly et al., 2013; Jaiswal, 2014; Mitsumoto et al., 2014; Wang et al., 2014), and others can be considered secondary in the development of ALS (Vucic and Kiernan, 2007).

#### Glutamate Excitotoxicity

Glutamate has a neurotoxic effect when it accumulates at the synapses (Gazulla and Cavero-Nagore, 2006). In patients with ALS (both spinal cord and motor cortex involvement), and in the superoxide dismutase 1 (SOD1) transgenic mouse model, a decrease in glutamate receptors (GluRs) was found in astrocytes. This decrease induces extracellular glutamate accumulation, which causes overstimulation of GluRs and neuronal death via excitotoxicity (Lin et al., 1998; Trotti et al., 1999; Barbeito et al., 2004; Pratt et al., 2012). In ALS patients, a decrease in glutamate transporters is mainly due to an alteration in messenger RNA (Lin et al., 1998; Honig et al., 2000).

#### Structural and Functional Abnormalities of the Mitochondria

Mitochondrial function disturbances, such as fragmentation and aggregation, are frequently found in ALS patients (Vielhaber et al., 2000; Chung and Suh, 2002; Krasnianski et al., 2005; Echaniz-Laguna et al., 2006; Crugnola et al., 2010; Cozzolino and Carrì, 2012). Increased crests, swelling, and fragmentation have been observed in the mitochondria of the spinal motor neurons and proximal axons of skeletal muscle in ALS-related tissues (Chung and Suh, 2002; Echaniz-Laguna et al., 2002; Boillée et al., 2006a). The increase in the misfolded SOD1 enzyme in the mitochondria of the spinal cord of mice is considered to be the main cause of mitochondrial dysfunction. In addition, aggregates of the enzyme SOD1 may also interact with the apoptosis regulator protein Bcl-2, inducing an apoptotic cascade and contributing to the deterioration of neurons and neuro-muscular degeneration (Boillée et al., 2006a).

#### Impaired Axonal Structure and Transport Defects

Axonal transport (retrograde and anterograde) is impaired in ALS patients and in mutant SOD1 mice, as evidenced by the accumulation of altered structures, such as mitochondria, neurofilaments, and autophagosomes, in the spinal motor neuron axons (Ikenaka et al., 2012; Zarei et al., 2015). Mutations in the dynein genes have been seen in models of ALS mice. Dyneins are responsible for the transport of mitochondria and autophagosomes, causing both to accumulate in the axon (Ikenaka et al., 2012). Autophagosomes are necessary for the elimination of altered mitochondria and dilated endoplasmic reticules, which accumulate in the axons of motor neurons and cause them to malfunction (Zarei et al., 2015). All of the above suggest that an alteration in axonal transport could be fundamental for the development of ALS.

#### Free Radical-Mediated Oxidative Stress

Increased free radical and oxidative damage has been found in biopsies from ALS patients, as well as in cerebrospinal fluid, serum, and urine samples (Simpson et al., 2004; Shin Hee and Lee Keun, 2013). This oxidative damage also affects RNA, which has been shown in both human central nervous system (CNS) biopsies and in mouse ALS models of SOD1 (Chang et al., 2008). The enzyme SOD1 is an important anti-oxidant. The alterations in the redox reactions are one of the first theories of how mutations in SOD1 can cause cytotoxicity (Carrì et al., 2015). In addition, it has been observed in the motor cortex, using positron emission tomography (PET), that increased oxidative stress is related to the severity of the disease in ALS patients (Tsujikawa et al., 2015).

#### Protein Aggregates

It has been shown that in ALS, abnormal protein accumulations are produced that aid in the pathogenesis of the disease (Blokhuis et al., 2013). The ubiquitin–proteasome (UP) system, which repairs and removes proteins, plays an important role in ALS, with ubiquitin-reactive inclusions being characteristic of this pathology (Han-Xiang et al., 2011; Pratt et al., 2012). Among these, the inclusions of proteins TDP-43 and p62 are indicative of this pathology (Al-Sarraj et al., 2011; Williams et al., 2016). Some ALS patients present numerous inclusions positive for p62 but negative for TDP-43 in the hippocampus and cerebellum (Al-Sarraj et al., 2011). These p62-positive and TDP-43-negative inclusions have also been observed in other CNS areas including the retina (Brettchneider et al., 2013; Fawzi et al., 2014).

#### Neuroinflammation

Neuroinflammation occurs in many neurodegenerative diseases, such as Parkinson’s, Alzheimer’s, and ALS, resulting in the activation of astroglial and microglial cells (Ramírez et al., 2017). Specifically, in ALS, it has been observed that reactive glia (microglia and astrocytes) can influence the damage and subsequent death of motor neurons (Vargas and Johnson, 2010; Philips and Robberecht, 2011; Liao et al., 2012). In the ALS, the presence of mutant proteins (SOD1 and TDP-43), oxidative stress, mitochondrial damage, etc., produces continuous damage, which can trigger a chronic activation of glial cells and, therefore, a sustained inflammatory process that could exacerbate neuronal damage (Philips and Robberecht, 2011). It has been observed that the mutant SOD1 protein can have a toxic function on motor neurons only when the activated microglia is present through a mechanism involving a toll receptor (CD14-TLR) that causes an increase in free radicals (Zhao et al., 2010). Astrocyte activation (Vargas and Johnson, 2010), microglial activation (Alexianu et al., 2001), and lymphocyte appearance (Engelhardt et al., 1993) have been found in animal models of ALS (mutants of SOD1) (Alexianu et al., 2001; Boillée et al., 2006b) and in ALS patients (Kushner et al., 1991; Kawamata et al., 1992; Nagy et al., 1994; Schiffer et al., 1996; Turner et al., 2004). In both cases, the mutant SOD1 causes the microglia to increase the expression of pro-inflammatory cytokines, such as IL-1β and TNF-α (Weydt et al., 2004), and inflammatory mediators, such as cyclooxygenase 2 (COX-2) (Almer et al., 2001) and nitric oxide (NO). The NO released by the microglia could induce apoptosis in motor neurons through the activation of Fas via (Raoul et al., 2002).

Mutant protein TDP-43 can produce microglial activation through the surface receptor CD14, which stimulates the NF-κB and AP-1 pathways and the inflammasome. This can cause a neurotoxic cascade leading to motor neuron death (Zhao et al., 2015). In the ALS SOD1 model, the microglia isolated at the onset of the disease has an M2 or anti-inflammatory phenotype; however, the microglia isolated at the end of the disease has a neurotoxic M1 phenotype. This demonstrates a dual role of microglial cells during the disease process in this ALS model (Liao et al., 2012).

Astrogliosis has been observed in ALS models with mutations in *C9orf72*, *FUS*, *SOD1*, and *TARDBP* genes (Wong et al., 1995; Liu et al., 2016; Sharma et al., 2016). In the last model, the death of the motor neurons could be due, in part, to a loss of the function of the TDP-43 protein in the astrocytes (Yang et al., 2014). In ALS SOD1 models, astrocytes can release ATP, causing the activation of microglial cells via purinergic receptors (P2X7) (Gandelman et al., 2010). They can also release transforming growth factor-β1 (TGF-β1), which can induce microglial inactivation, thereby eliminating the possible beneficial effects of the microglia and, thus, accelerating the progression of the pathology (Endo et al., 2015). Moreover, the microglia in ALS can affect astrocytes by favoring the appearance of a neurotoxic subtype (Liddelow et al., 2017).

Considering all of the above, both the microglia and reactive astrocytes can affect neural function in ALS by playing an important role in the progression of the disease.

### Genetics

Although most cases (90–95%) of ALS are sporadic (SALS) and not inherited, 10% of cases are of genetic origin (Byrne et al., 2011). Specific genetic locus mutations have been found to constitute the cases of FALS (Deivasigamani et al., 2014). The pattern of inheritance depends on the genes involved. Most cases are inherited in an autosomal dominant pattern (He et al., 2015). Men show more intense symptoms of the disease than women. There are many genes involved in the development of this disease. In FALS, mutations in the *C9orf72* gene represent 30–40% of cases, those in the *SOD1* gene make up 15–20%, those in the *FUS* and *TARDBP* genes each represent approximately 5% of cases, and the remaining genes that have been associated with FALS each represent a small proportion of cases (Pratt et al., 2012; Blokhuis et al., 2013). We next describe some of these genes.

#### *C9orf72* Gene

This gene is located at the locus 9p21 of chromosome 9. It has been proposed that the *C9orf72* mutation can decrease the C9orf72 protein, thus causing less endocytosis, which is necessary for autophagy (Mathis et al., 2019). Moreover, the massive accumulation of the expanded hexanucleotide GGGGCC could be neurotoxic and sequester proteins that bind to RNA, thereby causing the disruption of the machinery that processes RNA (Mathis et al., 2019). This mutation could also lead to increased vulnerability to excitotoxicity due to increased calcium permeability mediated by AMPA type receptors (Selvaraj et al., 2018). The form of inheritance is autosomal dominant, although some carriers do not develop the disease, so it has an incomplete penetrance (Renton et al., 2011). This variant represents the most frequent cause of SALS (7%) and FALS (30–40%) (Majounie et al., 2012). The manifestations at the onset of the disease are typical of ALS and have a bulbar onset. This variant is often associated with an earlier age of onset, a faster clinical course, and shorter survival (Schymick and Traynor, 2010).

#### *FUS* Gene

The *FUS* gene, located on the short arm of chromosome 16 (16p11.2), encodes a protein called sarcoma fusion protein (FUS) (Hübers et al., 2015; Scekic-Zahirovic et al., 2017). In ALS, mutations in the *FUS* gene have been observed, most of which can produce changes in amino acids in the region of the protein related to DNA binding and mRNA processing. These mutations may interfere with the importation of FUS into the cell nucleus, which will cause an accumulation of FUS in the cytoplasm. This has been observed particularly in the nerve cells that control muscle movement (Scekic-Zahirovic et al., 2017). This mutation occurs in 3–5% of FALS and in 1% of SALS cases (Deng et al., 2010; Mackenzie et al., 2010). In addition, patients with *FUS* mutations tend to develop the disease earlier and have a shorter life expectancy than those observed with mutations in other genes. Patients with ALS and *FUS* mutations may also develop frontotemporal dementia (FTD) (Kwiatkowski et al., 2009; Vance et al., 2009).

#### *OPTN* Gene

The optineurin gene (*OPTN*) is located on chromosome 10 and encodes a protein called optineurin, which is a multifunctional ubiquitin-binding phosphoprotein found in the cytoplasm. Changes of this protein can cause alterations in intracellular traffic and lead to inclusions in ALS. In addition, this protein is also involved in the signaling of the tumor necrosis factor α/NF-κB pathway (Zhu et al., 2007) and the mGluR (Ying et al., 2010). In 2010, Maruyama et al. (2010) and Schymick and Traynor (2010) found mutations in the *OPTN* gene in ALS patients. These mutations are present in 1.2% of patients with FALS (Del Bo et al., 2011). ALS patients with OPTN mutations presented typical spinal-onset disease (Weishaupt et al., 2013). Mutations in this gene were previously shown to be involved in primary open-angle glaucoma (Maruyama et al., 2010). This gene has also been linked to normotensional glaucoma in ALS patients (Weishaupt et al., 2013). In cells from patients with SALS and FALS, optineurin can be placed in inclusion bodies with TDP-43 (Maruyama et al., 2010), FUS (Ito et al., 2011), and SOD1 (Maruyama et al., 2010). Both retinal ganglion cells (RGCs) and motor neurons share common susceptibility factors (Weishaupt et al., 2013).

#### *SOD1* Gene

The *SOD1* gene is located on the long arm of chromosome 21 (21q22.11). This gene encodes a cytosolic enzyme called copper zinc (Cu/Zn) SOD1 (Gatchel and Zoghbi, 2005; Valentine et al., 2005) that plays a very important role in the elimination of superoxide radicals, thereby protecting against free radicals (Azadmanesh and Borgstahl, 2018). Around 2.5–23% of patients with FALS and 0.44–7% of those with SALS have mutations in SOD1 (Andersen, 2006; Van Es et al., 2010). Most of the inherited forms of SOD1 gene mutations are dominant (Hayward et al., 1998; Marucci et al., 2007). Mutations in the SOD1 enzyme can induce configurational changes in the SOD1 protein leading to motor neuron toxicity (Mathis et al., 2019). ALS patients who present mutations in SOD1 show an earlier time of onset and a longer duration of disease. In addition, they usually do not present cognitive disorders, and their motor symptoms usually begin in the lower extremities (Mathis et al., 2019).

#### *TARDBP* Gene

The Tar DNA binding protein (*TARDBP*) gene is located on the short arm of chromosome 1 (1p36.22) and encodes the TDP-43 protein. More than 40 mutations have been identified in the *TARDBP* gene and result in 6.5% of FALS cases. The form of inheritance is dominant (Kühnlein et al., 2008; Van Deerlin et al., 2008) and represents 0–5% of SALS cases (Kühnlein et al., 2008; Rutherford et al., 2008; Sreedharan et al., 2008). Most of the mutations cause changes in the amino acids in the TDP-43 protein and affect the region of the protein involved in the processing of RNA (Neumann et al., 2006; Da Cruz and Cleveland, 2011). Cytoplasmic inclusions of ubiquitin-reactive hyperphosphorylated TDP-43 proteins have been found in tissues of patients with FTD (Arai et al., 2006; Neumann et al., 2006). These inclusions have also been seen in the glial tissue and neurons of patients with SALS (Maekawa et al., 2009). These inclusions can often be placed with p62 and ubiquitin but are not found in FALS with *FUS* mutation (Vance et al., 2009) or *SOD1* mutation (Mackenzie et al., 2007). Some patients with FALS and *TARDBP* mutation may also develop FTD (Arai et al., 2006; Neumann et al., 2006).

## Materials and Methods

**FIGURE 1:**
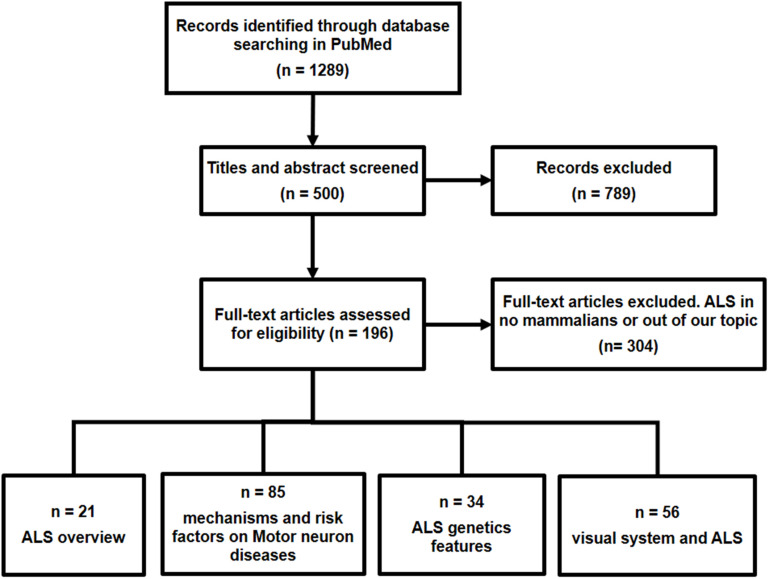
Flow chart materials and methods.

A literature search was performed up to May 2020 using the “MESH” terms in PubMed with the following keywords and word combinations: “Amyotrophic Lateral Sclerosis,” AND “Environmental Exposure,” “Nerve Degeneration,” “mitochondria,” “oxidative stress,” “Protein Aggregates,” “Microglia,” “Genetics,” “Oculomotor Muscles,” “Visual Pathways,” “Evoked Potentials, Visual,” “Contrast Sensitivity,” “Visual Fields,” “Visual Field Tests,” “Visual Acuity,” “Retina,” OR “Optical Coherence Tomography.” After filtering by author criteria (articles published in the last 10 years), English or Spanish language, and the condition that all address the relationship between ALS and visual pathway as the main subject, 196 articles were considered, and 304 articles did not satisfy the selection criteria (Figure 1).

*Inclusion criteria*: Articles were selected according to the following criteria: (i) articles focused on general features of ALS pathology, (ii) research that related the ALS with visual system alterations, (iii) retinal research developed in both ALS patients and animal experimental models of ALS, and (iv) articles based on human clinical trials with optical coherence tomography (OCT) analysis.

*Exclusion criteria*: Articles were excluded with the following characteristics: (i) research not carried out in mammalian experimental models of ALS and (ii) articles that did not have the sufficient outcomes associated with the objective of this review or did not meet the selection criteria of the authors.

## Amyotrophic Lateral Sclerosis and the Eye

As discussed above, ALS affects not only motor neurons, the spinal cord, the cerebellum, and large areas of the brain but also the visual system, including the oculomotor and visual pathways. However, patients usually do not have visual complaints. For this reason, studies focusing on the visual pathway are not common. Most previous studies were related to the oculomotor function and the study of visual evoked potentials (VEPs) that analyze the visual pathway. However, some studies of ALS have subsequently emerged that are more related to visual function, using tests such as visual acuity, contrast sensitivity, and visual field (VF). In addition, the retina has now been described as a “window to the brain,” and the changes that the brain suffers in neurodegenerative diseases can also appear in the retinal tissue (MacCormick et al., 2015). Changes in retinal tissue can be detected using OCT, which is a diagnostic technique widely used in ophthalmology and has recently been used for the analysis of retinal and optic nerve changes in neurodegenerative diseases such as ALS disease. This technique can help in the diagnosis and follow-up of these pathologies (Salobrar-García et al., 2015a,b, 2016).

**FIGURE 2:**
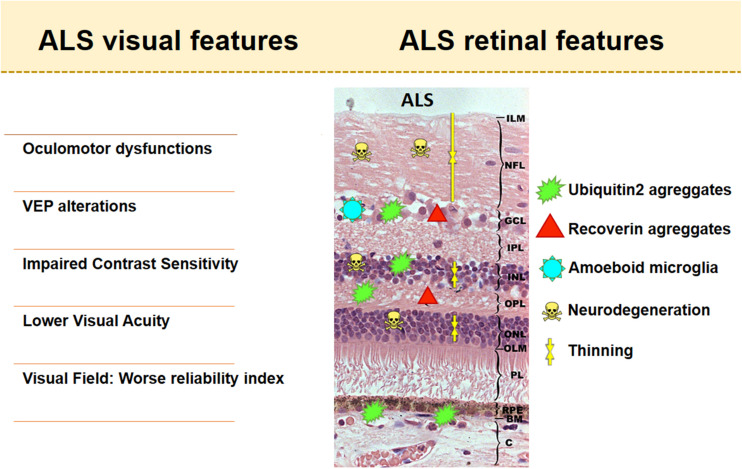
Visual and retinal features in amyotrophic lateral sclerosis (ALS). The retinal scheme represents the main histological findings found in retinas with ALS.

The following is a description of the main alterations found in this visual system using the aforementioned techniques and tests (Figure 2).

### Oculomotor Function Alterations

Amyotrophic lateral sclerosis is a condition that affects motor neurons and large areas of the brain, so ocular movements may be affected. Thus, numerous studies on oculomotor function have appeared since the 1980s and continue to appear today (Table 1 and Figure 2). Although oculomotor function is generally retained in ALS patients, they may manifest various oculomotor dysfunctions both at a relative early stage and in advanced stages of the disease. These include (i) a worsening of saccadic and pursuit eye movements (Leveille et al., 1982; Cohen and Caroscio, 1983; Saito and Yamamoto, 1989; Gizzi et al., 1992; Marti-Fàbregas and Roig, 1993; Ohki et al., 1994; Abel et al., 1995; Shaunak et al., 1995; Okuda et al., 2009; Donaghy et al., 2010; Moss et al., 2012; Kang et al., 2018); (ii) a lack of suppression of the vestibulo-ocular reflex (Ohki et al., 1994); (iii) a significant increase in error rates (distraction) and latency in anti-saccadic movements (Shaunak et al., 1995; Donaghy et al., 2010) that may evoke saccadic paradigms, head shaking (Kang et al., 2018), and positional nystagmus of central origin (Saito and Yamamoto, 1989; Marti-Fàbregas and Roig, 1993; Ohki et al., 1994; Kang et al., 2018); (iv) gaze fixation instability (Saito and Yamamoto, 1989; Palmowski et al., 1995; Shaunak et al., 1995; Donaghy et al., 2009; Moss et al., 2012; Kang et al., 2018); (v) eyelid opening apraxia (Averbuch-Heller et al., 1998; Moss et al., 2012); and (vi) square wave jerks (Gizzi et al., 1992; Shaunak et al., 1995; Kang et al., 2018), which may reflect the incidence of secondary abnormalities, such as parkinsonism (Gizzi et al., 1992), and facilitate prefrontal dysfunction in these patients (Shaunak et al., 1995). In bulbar onset compared with spinal onset, saccadic dysmetria and abnormal cogwheeling smooth pursuits are increased, which suggests neurodegeneration in ALS involving more than motor neurons (dysfunction of vestibule cerebellar connections), especially in bulbar-onset disease (Kang et al., 2018). In a longitudinal study (Proudfoot et al., 2016), ALS patients, even those with normal saccadic function, were shown to have problems with executive and visual search tasks. These impairments were often more severe than expected in ALS patients. However, no significant progression was observed in the longitudinal study, nor were changes found in the connectivity of the R-FMRI network (Proudfoot et al., 2016). In the postmortem histopathological examination of ALS patients, cell loss was found in the substantia nigra and the rostral interstitial nucleus of the medial longitudinal fasciculus, suggesting that the early involvement of vertical saccades might correspond to a different clinical–pathological type (Averbuch-Heller et al., 1998). Therefore, as suggested by Sharma et al. (2011), changes in the oculomotor function of ALS patients could be promising biomarkers for the mechanical diagnosis, prognosis, and follow-up of ALS.

**TABLE 1:** Previous studies of extraocular movements in ALS patients.

Study and year of publication	Type of study	Number of ALS/control patients	Disease duration (months)	ALSFRS score	Alterations in ocular movements in ALS
Leveille et al., 1982	–	10/–	–	–	↓Saccadic pursuit, unidirectional saccadic pursuit, ↓saccadic velocities
Saito and Yamamoto, 1989	Observational	23/100	–	–	Slight limitations of upward only, upward and downward and upward and horizontal gaze, incomplete convergence, horizontal gaze nystagmus. ↑Amplitude ratio of saccade and ↑degree of ocular dysmetria
Marti-Fàbregas and Roig, 1993	Cross-sectional	13/16	29.09	–	↑Saccadic latencies and ↓smooth pursuit gain. Positive relationship between smooth pursuit saccadic intrusions and the bulbar clinical score and the rate of progression and a ↓optokinetic nystagmus maximal velocity in patients with pseudobulbar syndrome
Shaunak et al., 1995	Cross-sectional	17/11	24.5 (6–60)	–	↑Error rates (distractibility) and latency in the anti-saccade and remembered saccade paradigms. ↑Square−wave jerks. Gaze fixation instability
Donaghy et al., 2010	Cross-sectional	30 spinal and 14 bulbar/45	52	35 (18–47)	↓Reflexive saccades in bulbar-onset vs. to spinal-onset patients and controls. ↑Anti-saccade latency and ↑anti-saccade type 1 errors. ↓“Proportion of time spent in smooth pursuit” and ↓smooth pursuit “velocity gain”
Moss et al., 2012	Cross-sectional	63/37	43.2 ± 37.25 (7.2–204)	16 (6–46)	Gaze impersistence, voluntary upgaze restriction, eyelid opening apraxia, saccadic horizontal pursuits
Ohki et al., 1994	Observational	9/–	13 (3–49)	Early stages	↓Velocity of saccades. Abnormalities of smooth pursuit, optokinetic nystagmus, and visual poor suppression of vestibular ocular reflect
Proudfoot et al., 2016	Cross-sectional/longitudinal	61/39	38.6 (31)	33.5 (6.5)	↓Executive and visual search tasks; normal basic saccadic function. ↓Anti-saccade performance, ↑error rate and latency
Kang et al., 2018	Retrospective/observational	22 spinal cord/10 bulbar	Spinal 55.1 ± 9.5, bulbar 62.2 ± 11.1	–	Square wave jerks, saccadic dysmetria, abnormal cogwheeling smooth pursuits, head shaking, and positional nystagmus of central origin. ↑ Abnormal smooth pursuits and saccadic dysmetria

*ALS, amyotrophic lateral sclerosis; ALSFRS, Amyotrophic Lateral Sclerosis Functional Rating Scale.*

The extraocular muscles (EOMs) and their motor neurons are retained in ALS. However, from the onset of this disease, the muscles of the limbs show axon retraction at the neuromuscular junctions. Wnt is a preserved family of secreted signaling molecules that are primarily involved in the formation of neuromuscular junctions. This signaling pathway was analyzed both in ALS patients and in a SOD1G93A mouse model for its possible implications in the preservation of normal morphology and the function in EOMs in this disease (McLoon et al., 2014). The authors found differential patterns of expression for Wnt1 and Wnt3a isoforms in the EOMs for limb muscles, especially at the neuromuscular junction level. This suggests that in ALS patients, this signaling pathway is preserved in the EOMs and dysregulated in the muscles of the limbs that subsequently develop a pathology (McLoon et al., 2014).

### Visual Pathway Alterations

The alteration of the visual pathway can be analyzed using VEPs, contrast sensitivity, and VF. Next, we describe the different alterations found in the visual pathway of each.

#### Visual Evoked Potentials

Few studies have analyzed the VEPs in ALS patients (Table 2 and Figure 2). It was not until 1986 that alterations in VEPs were found in patients with ALS, with abnormal relative difference in latencies between each eye. In this study of 32 patients analyzed, only four had VEP abnormalities (increased latency time); however, these alterations were mild (Matheson et al., 1986). In other studies, while wave latency and amplitudes were within normal limits in all ALS patients (Ghezzi et al., 1989; Palma et al., 1993), somatosensory evoked potentials were abnormally delayed (N9–N13 and N13–N19 latencies), but no correlation was found among these abnormalities and the duration and severity of the disease (Ghezzi et al., 1989).

**TABLE 2:** Psychophysics visual test studies in ALS patients.

Study and year of publication	Alterations in visual functions in ALS				
	VEP	ERG	VA	VF	CS
Matheson et al., 1986	Abnormal latencies between eyes	–	–	–	–
Ghezzi et al., 1989	WNL	–	–	–	–
Münte et al., 1998	P300 delayed and attenuated	P1 absent	–	–	–
González Díaz et al., 2004	P100 latency prolongation	WNL	–	–	–
Fawzi et al., 2014	–	–	–	–	↓CS
Volpe et al., 2015	–	–	No changes high contrast VA	–	No changes
Moss et al., 2016	–	–	↓High contrast and low contrast VA	–	–
Liu et al., 2018	–	–	–	MS↓ and ↑sLV	–
Rojas et al., 2019	–	–	No changes high contrast VA	↓ reliability index (↑fixation losses, ↑false positives and negatives)	–

*ALS, amyotrophic lateral sclerosis; VEP, visual evoked potentials; ERG, electroretinogram; VA, visual acuity; VF, visual field; CS, contrast sensitivity; WNL, within normal limits; MS, mean sensibility; sLV, square of loss variance.*

In addition, in ALS patients the P1 component was also found to be absent, and the P300 component was delayed and attenuated (Münte et al., 1998). In a study of six ALS patients, only one patient with a 9-month disease evolution had abnormal P100 bilateral extension VEPs and a significant interocular difference of P100. In addition, none of the patients presented alterations in their electroretinogram (González Díaz et al., 2004). Subsequently, electrophysiological studies of ALS patients showed new evidence of cortical participation involving visual areas, with alterations in the early sensory components of VEPs (Münte et al., 1998).

#### Contrast Sensitivity

While one study revealed impaired contrast sensitivity during an eye examination in two ALS patients with *C9orf72* mutation (Fawzi et al., 2014), another study concluded that this function is not affected in ALS patients (Volpe et al., 2015; Table 2 and Figure 2).

#### Visual Acuity

Visual acuity in ALS patients is controversial. While a study found a lower visual acuity in ALS patients in both high-contrast and low-contrast (2.5% and 1.5%) visual acuity with Sloan charts (Moss et al., 2012), in others, the visual acuity exam revealed no differences in monocular high-contrast visual acuity (Volpe et al., 2015; Moss et al., 2016; Rojas et al., 2019) or low-contrast visual acuity (Moss et al., 2016).

#### Visual Field

Only two studies describe VF in early ALS spinal onset patients. With a decrease of mean sensitivity and an increase of the square of loss variance (Liu et al., 2018), ALS patients presented a worse reliability index (fixation losses, false positives, and negatives) due to motor difficulties. For this reason, the authors suggested that VF is not a suitable test to assess ALS patients (Rojas et al., 2019; Table 2 and Figure 2).

### Retinal Abnormalities in Amyotrophic Lateral Sclerosis

Retinal tissue can be analyzed using histological techniques on postmortem tissues or using the OCT technique mentioned above. This technique is an optical analog of ultrasonic imaging using low-coherence interferometry to produce cross-sectional images of the retina and allows *in vivo* observation of retinal tissue alterations (Bhende et al., 2018).

#### Retinal Histopathological Studies in Amyotrophic Lateral Sclerosis Patients and Amyotrophic Lateral Sclerosis Experimental Models of Mammals

Few studies have focused on the histopathology of retinal tissue in both ALS patients and animal models of mammals with this condition. Histopathological studies in ALS patients demonstrated intraretinal protein inclusions. The first histopathological analysis of the retinas of patients with ALS was performed in 2014 on a patient with the *C9orf72* mutation. In this study, the authors found p62-positive and pTDP43-negative intracytoplasmic perinuclear inclusions in the inner nuclear layer (INL). These deposits were similar to those observed in the dentate gyrus of ALS patients with the *C9orf72* mutation. The p62-positive staining was colocalized for both the poly-(GA)n dipeptide repeat and ubiquitin in the retina, which is similar to the perinuclear inclusions located in the brains of patients with this mutation. The p62-positive inclusions found were likely located in specific cone bipolar cells and within the amacrine and horizontal cells, as they were also marked with GLT-1 and recoverin. The authors suggested that these deposits may be related to the affectation of contrast sensitivity (Fawzi et al., 2014). In addition, in other ALS patients with *C9orf72* mutations, specific p62 inclusions were observed in the retinal ganglion cell layer (GCL) in a far smaller proportion than in INL (94.9% in INL vs. 5.1% in GCL). Numerous positive ubiquitin2 + aggregates were also observed in a mutant *UBQLN2* transgenic mice experimental model, mainly in the INL, with fewer in the outer plexiform layer (OPL) and some in the GCL (Volpe et al., 2015). The accumulation of ubiquitin2 aggregates in the layers of the retina with more synapses is related to the accumulation of these aggregates in the dendritic spines of the hippocampus. The location of the aggregates at the synapses, and their spines, may be associated with the dementia observed in this experimental model of ALS. In addition, few ubiquitin2 positive aggregates were detected in the subretinal space of the retinal pigment epithelium, which sometimes elevated these levels in the same way as druses (Volpe et al., 2015). Furthermore, lipofuscin deposits sometimes related to subretinal drusen-like aggregates were found in progranulin-deficient FTD patients (Ward et al., 2017). Retinal thinning in these patients was detected by OCT before symptoms, suggesting that the eye is affected in progranulin-deficient FTD disease (Ward et al., 2014).

As noted previously, microglial and astroglial cell activation occurs in ALS. However, to our knowledge, there are only two works that analyzed the glial cells of the retina in relation to ALS. hSOD1 + vacuoles located in the dendrites of excitatory retinal neurons were observed in a mouse model of ALS SOD1 (*SOD1^*G*93*A*^*), mainly in the inner plexiform layer (IPL) and rarely in the GCL and INL. However, there were no signs of activation of either the astroglia or the microglia of the retina compared with those of wild-type mice (Ringer et al., 2017). In contrast, microglial activation was demonstrated in a mouse model of ALS [devoid of ran-binding protein2 (Ranbp2)]. Ranbp2 is a protein involved in nucleo-cytoplasmic transport whose regulation is impaired in both SALS and FALS (Ferreira, 2019). In ALS mice without this protein (with respect to wild-type controls), there was an increase in the number of CD45 +, CD11b +, and F4/80 + microglial cells surrounding the RGCs, in addition to a notable increase in amoeboid forms. An increase of metalloproteinase 28, which is an immune regulator, was also observed in the RGCs, suggesting that Ranbp2 may be involved in the signaling between the microglia and the RGCs in the immune response of ALS disease (Cho et al., 2019; Figure 2).

#### Optical Coherence Tomography

Although the first study performed in 75 ALS patients with Cirrus OCT showed no changes in the peripapillary and macular areas (Roth et al., 2013), further studies have shown that there are changes in the retina of ALS patients (Ringelstein et al., 2014; Volpe et al., 2015; Hübers et al., 2016; Simonett et al., 2016; Mukherjee et al., 2017; Abdelhak et al., 2018; Liu et al., 2018; Rohani et al., 2018; Rojas et al., 2019; Table 3).

**TABLE 3:** OCT studies in ALS patients.

Study and year of publication	OCT device	Type of study	Number of ALS/control patients	Disease duration (months)	ALSFRS-R score	Alterations in visual functions	Differences ALS vs. control						Correlation OCT-ALSFRS-R
							Macula	GCC	pRNFL	mRNFL	ONL	INL	
Roth et al., 2013	Cirrus HD-OCT	Cross-sectional	76/54	42 ± 34 (7–166)	34 ± 7	–	–	No	No	No	No	No	No
Ringelstein et al., 2014	SD-OCT Spectralis	Cross-sectional	24/24	22.3 ± 22.57 (3–120)		–	–	No	↓	↓	No	↓	No
Hübers et al., 2016	SD-OCT Spectralis	Cross-sectional	71/20	12 (2–98)	40 (16–48)	–	–	No	↓	No	No	↓	–
Volpe et al., 2015	SD-OCT Spectralis	Cross-sectional	16/15	85.3 ± 110.79	–	= VA, color vision, and CS. Histopathology study	–	–	↓	↓		–	No. OCT inversely correlates with disease duration
Simonett et al., 2016	SD-OCT Spectralis	Cross-sectional	21/21	43.2 ± 43.4 (10–197)	28.1 ± 12.5	–	–	No	–	↓	No	No	No. (correlation FVC% and FEV1%)
Mukherjee et al., 2017	SD-OCT Spectralis	Cross-sectional	21/normative OCT database	–	30 ± 10	VA (Snellen)	–	–	↓Global and six sectors	–	–	–	No
Rohani et al., 2018	SD-OCT Topcon 3D	Cross-sectional	20	14.5 ± 11.3	33.1 ± 3.8	–	–	–	↓Mean, sup and nasal	–	–	–	Direct correlation
Liu et al., 2018	HD-OCT Cirrus 4000	Cross-sectional	51/126	18.46 ± 6.16 (6–72)	39.58 ± 10.41 (10–48)	VF: MS↓ and ↑sLV	No	No	↑Nasal quadrant	–	–	–	Macula direct correlation (temporal Q correlated with duration of the disease)
Abdelhak et al., 2018	SD-OCT Spectralis	Cross-sectional	34/20	12 (7–17)	–	Diameters of retinal vessels	–	No	No	–	↓	No	Retinal thickness inverse correlation
Rojas et al., 2019	HD-OCT Cirrus 4000	Prospective longitudinal	38/20	10.80 ± 5.5 (1–18)	Baseline 29.50 ± 14.89 Follow-up 35.6 ± 14.08 (some patients dead)	VA and VF	↑Temporal and ↑inferior. Follow-up: ↓ Inferior	No	↓Sup, ↓inf. ↓H3, H5, H6, H12 ↑H8	–	–	–	pRNFL inverse correlation

*ALS, amyotrophic lateral sclerosis; GCC, ganglion cell complex; RNFL, retinal nerve fiber layer; pRNFL, peripapillary RNFL; mRNFL, macular RNFL; ONL, outer nuclear layer; INL, inner nuclear layer; VA, visual acuity; CS, contrast sensitivity; FVC%, Forced vital capacity% predicted; FEV1%, forced expiratory volume in 1 s% predicted; MS, mean sensibility; sLV, square of loss variance; ALSFRS, Amyotrophic Lateral Sclerosis Functional Rating Scale.*

In the retina of ALS patients, high-resolution SD-OCT revealed reduction in the mean total macular thickness, in the peripapillary retinal nerve fiber layer (pRNFL), the INL (Ringelstein et al., 2014; Hübers et al., 2016), and the outer nuclear layer (ONL) (Abdelhak et al., 2018), suggesting neurodegeneration of the retina in ALS patients.

Volpe et al. studied whether clinical and histopathological findings were present in the eyes of ALS patients and explored their correlation with an animal model (ALS/dementia transgenic mice with dysfunctional ubiquilin2, UBQLN2P497H) (Volpe et al., 2015). Using SD-OCT, the authors observed that (i) in ALS patients compared with the controls, there was a decrease in total macular volume; (ii) 37.5% of ALS patients showed an average pRNFL below the first percentile, and temporal and papillomacular bundle were more affected; and (iii) in ALS patients, the total macular thickness and pRNFL thickness correlated inversely with the time of evolution of ALS (Volpe et al., 2015).

Recently, in a study performed in early ALS patients with spinal onset and without ocular diseases, a significant macular thickness increase was found in the temporal and inferior areas of the inner macular ring in comparison with that in a healthy control, suggesting that this retinal thickening in early ALS patients could be due a microglial activation in the neuroinflammatory process (Ringelstein et al., 2014; Rojas et al., 2019). In contrast, in another study in ALS patients, only the macular retinal nerve fiber layer (mRNFL) showed a significant thickness decrease by OCT, which correlated positively with pulmonary function tests (Simonett et al., 2016). However, neither total macular thickness nor macular thickness showed changes (Simonett et al., 2016).

Only one study exists that detected a thinning in the ONL (Abdelhak et al., 2018), suggesting a possible impact on the photoreceptors and linking this finding with this subclinical visual acuity impairment (Moss et al., 2012; Abdelhak et al., 2018). Using SD-OCT, this work analyzed the retinal vessels, finding that in ALS patients compared with the control, the outer wall thickness of the retinal vessels was higher than in the control group (Abdelhak et al., 2018). Similar microvascular alterations in the brain and spinal cord of ALS model mice were found to precede the degeneration of motor neurons (Zhong et al., 2008).

In ALS patients, an analysis of the relationship of clinical features and retinal changes using SD-OCT and diffusion tensor imaging (DTI) found no significant correlation between clinical features and retinal thickness; however, there was a direct correlation between retinal thickness and fractional anisotropy of the corticospinal tract (Hübers et al., 2016). On the basis of these observations, it was suggested that retinal changes could be related to damage of white matter in the corticospinal tract and may be a possible biomarker in ALS (Hübers et al., 2016).

Although several papers have demonstrated the pRNFL decrease in ALS patients without ocular pathology (Mukherjee et al., 2017; Rohani et al., 2018; Rojas et al., 2019), there is controversy about its correlation with ALSFRS-R values. While one study did not demonstrate a correlation between RNFL thickness and the ALSFRS-R score and their progression rates (Mukherjee et al., 2017), other authors found a correlation with some OCT parameters (Abdelhak et al., 2018; Liu et al., 2018; Rohani et al., 2018; Rojas et al., 2019; Salobrar-García et al., 2019). In a study of the pRNFL thickness in four quadrants, the average pRNFL thickness showed a significant positive correlation with the ALSFRS-R score (Rohani et al., 2018), and the pRNFL thickness in the inferior sector was negative (Rojas et al., 2019). When the analysis was more detailed, dividing the papilla into 12 hourly sectors, sectors H5 and H6 had a positive direct significant correlation, and H8 had an inverse significant correlation of pRNFL with the ALSFRS-R values (Rojas et al., 2019). In addition, the entire retinal thickness correlated negatively with the ALSFRS-R score (Abdelhak et al., 2018).

Follow-up OCT studies of ALS patients are very rare. In the only one performed of early ALS patients with spinal onset (basal patients) who were examined 6 months after the basal scan (follow-up patients), the SD-OCT follow-up analysis of ALS patients showed a significant macular thickness decrease in the inferior areas of the inner and outer macular ring and a significant pRNFL thickness decrease in the superior and inferior quadrants, compared with the baseline (Rojas et al., 2019).

Asymmetry is a typical hallmark of ALS, as mentioned above. In the left eye (LE) of ALS patients, after adjustment for multiplicity, there was a significant decrease in pRNFL thickness in the nasal quadrant compared with the corresponding quadrant thickness in the right eye (RE) (Rohani et al., 2018). In the follow-up study mentioned above (Rojas et al., 2019), there were significant interocular asymmetries in some areas between the LE, which were always thinner, and the RE were observed. In the baseline ALS group, in the LE, the inferior-nasal quadrant of the macular ganglion cell complex (GCC), and the H7 and H9 hourly-sectors of the pRNFL were significantly thinner than those in the RE, while in the follow-up ALS group, both the supero-nasal quadrant of macular GCC and the temporal quadrant, and the H8 and H9 hourly sectors of the pRNFL, were significantly thinner in the LE than those in the RE. Therefore, the asymmetric participation of the CNS in this disease is not exclusive to the motor system (Rohani et al., 2018).

Thinning of the inner retina was also observed by SD-OCT in other neurodegenerative diseases, including Alzheimer’s disease and Parkinson’s disease, in which both RNFL and GCL thinning were detected (Moreno-Ramos et al., 2013; Coppola et al., 2015; Salobrar-García et al., 2015b; Garcia-Martin et al., 2016). However, in FTD, OCT identified ONL thinning, specifically in the ellipsoid zone, and this thinning of the outer retina correlated with cognitive changes (Kim et al., 2017). Moreover, the relationship between ONL thinning and FTD was especially apparent in the subgroup of patients considered to have “likely tauopathy” based on their symptoms or their genetics (Kim et al., 2017).

The differences observed in retinal thickness measurements using OCT may be due to the following: (i) the different disease stages of ALS patients included in the studies; (ii) the heterogeneous nature of this pathology (Hübers et al., 2016); and (iii) the small number of participants in these studies, since most ALS patients do not manifest visual problems.

Many authors observed changes in retinal thickness, as shown in this section. Therefore, retinal changes constitute a biomarker of neurodegeneration and progression of ALS disease (Hübers et al., 2016; Rohani et al., 2018), and OCT analysis is a useful tool for the study of this pathology (Rojas et al., 2019).

## Conclusion

In view of the above, changes in visual function are moderate in ALS. The main changes occur at the oculomotor level, but with no great affect. Functional tests such as VA, contrast sensitivity, VF, and VEPs show mild alterations in ALS. However, the low rate of functional impairment does not mean that there are no structural changes in the visual pathway in ALS patients. New techniques such as OCT have made it possible to detect structural changes in the retina (due to retrograde or anterograde involvement), but which ultimately reflect changes in the visual pathway. These changes monitored with OCT could help to follow up this pathology. However, these studies are recent and are not homogeneous due to differences in the patients’ degree of disease, time of evolution, age group, or ALS scores. In addition, differences in the OCT technology make it difficult to compare between the different studies. Thus, it is necessary to perform more studies to analyze the retinal changes that occur in ALS disease. In addition, there are also many unanswered questions, including when and where the changes in the retina first occur, which subgroups of patients exhibit retinal phenotypes, and whether these phenotypes change over time. Longer studies are needed to determine whether retinal thickness might be a useful way to measure disease progression and whether patients with different genetic mutations that cause ALS experience more severe retinal involvement.

We conclude that, in addition to CNS disorders, there are also peripheral neurological diseases that can affect the retina. We have shown that the retina has great sensitivity as a biomarker for susceptibility/risk in MND, despite the fact that the retinal changes do not produce clinical visual symptoms. Therefore, the use of OCT in ALS patients could be a recommended test within the diagnostic techniques of this pathology.

## Author Contributions

PR, AR, IL-C, ES-G, RH, and JR designed the concept and drafted the manuscript. JF-A, IL-C, ES-G, MC, LE-H, and JS collected the literature, analyzed the data, and edited the language in the manuscript. All authors contributed to the article and approved the submitted version.

## Conflict of Interest

The authors declare that the research was conducted in the absence of any commercial or financial relationships that could be construed as a potential conflict of interest.
